# Noscapine Acts as a Protease Inhibitor of In Vitro Elastase-Induced Collagen Deposition in Equine Endometrium

**DOI:** 10.3390/ijms22105333

**Published:** 2021-05-19

**Authors:** Ana Amaral, Carina Fernandes, Anna Szóstek-Mioduchowska, Maria Rosa Rebordão, Dariusz Jan Skarzynski, Graça Ferreira-Dias

**Affiliations:** 1 CIISA—Centro de Investigação Interdisciplinar em Sanidade Animal, Departamento de Morfologia e Função, Faculdade de Medicina Veterinária, Universidade de Lisboa, 1300-477 Lisboa, Portugal; nita.amaral@gmail.com (A.A.); fachica@hotmail.com (C.F.); milorebordao@gmail.com (M.R.R.); 2 Institute of Animal Reproduction and Food Research, Polish Academy of Science, 10-748 Olsztyn, Poland; a.szostek-mioduchowska@pan.olsztyn.pl (A.S.-M.); d.skarzynski@pan.olsztyn.pl (D.J.S.); 3 Polytechnic of Coimbra, Coimbra Agriculture School, Bencanta, 3045-601 Coimbra, Portugal

**Keywords:** equine, endometrosis, fibrosis, elastase, noscapine, inhibition, collagen

## Abstract

Endometrosis is a reproductive pathology that is responsible for mare infertility. Our recent studies have focused on the involvement of neutrophil extracellular traps enzymes, such as elastase (ELA), in the development of equine endometrosis. Noscapine (NOSC) is an alkaloid derived from poppy opium with anticough, antistroke, anticancer, and antifibrotic properties. The present work investigates the putative inhibitory in vitro effect of NOSC on collagen type I alpha 2 chain (*COL1A2*) mRNA and COL1 protein relative abundance induced by ELA in endometrial explants of mares in the follicular or mid-luteal phases at 24 or 48 h of treatment. The *COL1A2* mRNA was evaluated by qPCR and COL1 protein relative abundance by Western blot. In equine endometrial explants, ELA increased COL 1 expression, while NOSC inhibited it at both estrous cycle phases and treatment times. These findings contribute to the future development of new endometrosis treatment approaches. Noscapine could be a drug capable of preventing collagen synthesis in mare’s endometrium and facilitate the therapeutic approach.

## 1. Introduction

One of the main reproductive pathologies esponsible for equine infertility is equine endometrial fibrosis, also known as endometrosis [1]. Altered endometrial glands with periglandular and/or stromal endometrial fibrosis [2] characterize the occurrence of fibrotic and degenerative changes in mare endometrium [3]. It is an important cause of early embryonic loss since the endometrial glands are gathered in nests filled with cysts [4].

For the last years, some putative pathways that trigger endometrosis have been studied. The role of some inflammatory mediators, such as prostaglandins, transforming growth factor β1, and interleukins have already been investigated [5,6,7,8]. Moreover, our recent work has been focused on the involvement of neutrophil extracellular traps (NETs) enzymes in the development of equine endometrosis, such as elastase (ELA), cathepsin G (CAT) and myeloperoxidase (MPO) [9,10,11,12,13,14]. At post-breeding endometritis, a fast physiological neutrophil influx to the uterus occurs [15,16] to eliminate the excess of spermatozoa, contaminating bacteria, and debris [17,18]. Afterwards, neutrophils release their DNA and cytoplasm enzymes to the extracellular environment in a way of enhancing the innate immune response [19,20]. Conversely, NETs enzymes may also drive a profibrotic reaction in mare endometrium that increases the endometrial production of collagen type I (COL1) [9,11,12,13,14].

However, endometrosis was also found in aged maiden mares that were not exposed to semen [2,21]. In fact, not only semen induces an endometrial response, but also the presence of urine, bacteria, fungi or yeasts in the uterus are capable of inducing endometritis [15,16]. In addition, age [22], poor vulvar conformation, pneumovagina, pendulous uterus, cervical fibrosis [23,24,25,26], and degenerative alterations [24] have been reported as triggering factors of persistent endometritis culminating in endometrosis establishment. It can be suggested that a permanent collagen synthesis in mare endometrium could be activated by local infiltration of immune cells, as an alteration of inflammation and endometrial repair balance associated to a genetic predisposition [27].

Elastase is a serine neutrophil protease that acts by degrading components of the extracellular matrix as collagen, elastin, and fibronectin [28]. In NETs, the dominant proteolytic activity is attributed to ELA [29]. Additionally, this protease has been largely studied because of its involvement in the development of fibrosis. In human lung fibrosis, ELA was linked to the disease progression once it stimulated myofibroblast differentiation and induced in vitro lung fibroblast proliferation [30]. Moreover, in human cystic fibrosis, ELA was also demonstrated to be involved in the pathogenesis and severity of the disease [31]. More recently, our team has found the involvement of ELA in endometrosis development [9,11,12], and that its inhibition by sivelestat sodium salt reduced in vitro COL1 production by equine endometrial explants [12].

During the last decades, the use of dimethyl sulfoxide [32], kerosene [33], or mesenchymal stem cells [34,35] were proposed to treat mare endometritis and/or endometrosis. Unfortunately, none of the proposed treatments revealed themselves as effective on endometrial fibrosis reduction, nor increased fertility rates. Interestingly, our latest in vitro studies in equine endometrial explants showed that selective inhibitors of NETs enzymes could reduce COL1 expression [9,11,12,13,14]. Sivelestat sodium salt, β-keto-phosphonic acid, and 4-aminobenzoic acid hydrazide are selective ELA, CAT and MPO inhibitors, respectively, which revealed capability of reducing the COL1 induced by these enzymes [11,12,13,14]. These new findings might be the grounds for future development of drugs to be used on the prophylaxis or therapy of mare endometrosis. Although, the complexity of this disease proposes that effective therapeutic interventions might need the administration of a single inhibitor capable of inhibiting both ELA, CAT and MPO.

When compared to CAT or MPO, ELA was the NETs’ enzyme that increased COL1 the most, revealing its importance as a profibrotic agent of endometrosis [9]. Therefore, the effective inhibition of ELA will be an important step for endometrosis treatment. Although, the inhibition of individual triggering factors of endometrosis may fail the objective of reducing collagen deposition, as other agents could also exert profibrotic effects. Nevertheless, finding a molecule that acts in a specific key point of the fibrosis pathway will result in a blockade of the fibrotic process, regardless of the causing agent.

Noscapine is an alkaloid derived from poppy opium with its first antitussive application in 1930 [36,37]. Later, NOSC was also discovered to act as an antistroke drug, reducing stroke sequelae and mortality [38,39]. After a focal ischemia, the brain released bradykinin, and NOSC treatment was able to antagonize bradykinin receptors [39]. Additionally, NOSC has showed anti-inflammatory properties through cytokine regulation. Its action on delaying tubulin dynamics may reduce the transduction signal and impair protein transcription [40]. Moreover, NOSC was reported to decrease the regulation of mediators of inflammation in renal ischemia-reperfusion injury in rats by inhibiting bradykinin receptors [39]. Noscapine also acts as an anti-neoplasic agent both in vitro and in a mouse in vivo model, at higher doses (20–120 mg/kg) [41] than those used as an antitussive drug (1 mg/kg) [42]. In fact, NOSC revealed to be an effective drug against several types of cancer in studies using cell lines or mice models [37,43,44]. Furthermore, NOSC has also been described as an antifibrotic drug, even though few studies have been performed on NOSC antifibrotic properties. In a mouse model of bleomycin-induced pulmonary fibrosis, NOSC inhibited fibrosis progression [45]. Noscapine also acts as a protease inhibitor. Kumar et al. [46] characterized the molecular binding of NOSC to the main protease in severe acute respiratory syndrome coronavirus 2 that causes COVID-19 disease.

Thus, we have decided to investigate if NOSC inhibits the profibrotic effect of NETs enzymes involved in equine endometrosis development. We hypothesized that NOSC inhibits proteases profibrotic effect in endometrial explants in a nonselective way. Therefore, the aim of this study was to evaluate the in vitro inhibitory effect of NOSC on ELA-induced collagen formation in mare endometrial explants by assessing *COL1A2* mRNA and the relative abundance of COL1 protein, at different estrous cycle phases and treatment times.

## 2. Results

### 2.1. Viability Data of Cultured Equine Endometrial Explants

The results of lactate dehydrogenase (LDH) activity after 1, 24 or 48 h are presented in Table 1. The activity of LDH at 24 h (*p* < 0.05) and 48 h (*p* < 0.01) differed from 1 h of incubation. The comparison between 24 and 48 h incubation also showed statistical significance (*p* < 0.05). The LDH activity data were independent of estrous cycle phases.

**Figure 1:**
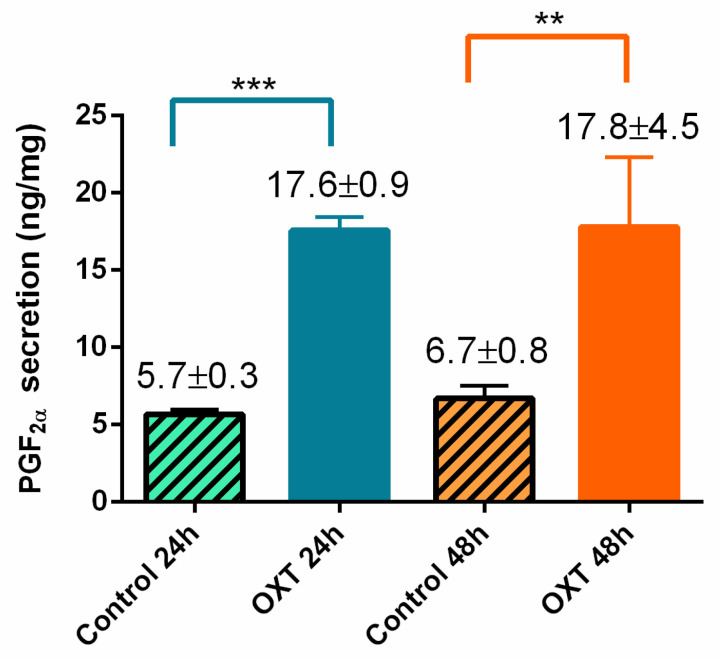
Equine endometrial explant secretion of prostaglandin (PG)F_2α_ treated with oxytocin (OXT; 10−7 M) for 24 or 48 h. Secretion of PGF_2α_ (ng/mL) is presented as mean ± SEM. Results were considered significant at *p* < 0.05. Asterisks represent statistical differences between OXT treatment and the respective control within the treatment time (** *p* < 0.01; *** *p* < 0.001).

Prostaglandin (PG) F_2α_ data are presented in Figure 1 and were independent of estrous cycle phases. Prostaglandin F_2α_ secretion increased when challenged with oxytocin (OXT) treatment both at 24 (*p* < 0.001) and 48 h (*p* < 0.01). No differences were found between 24 and 48 h of treatment.

### 2.2. Noscapine Reduced the ELA-Induced COL1 Expression in Both Estrous Cycle Phases and Treatment Times

**Figure 2:**
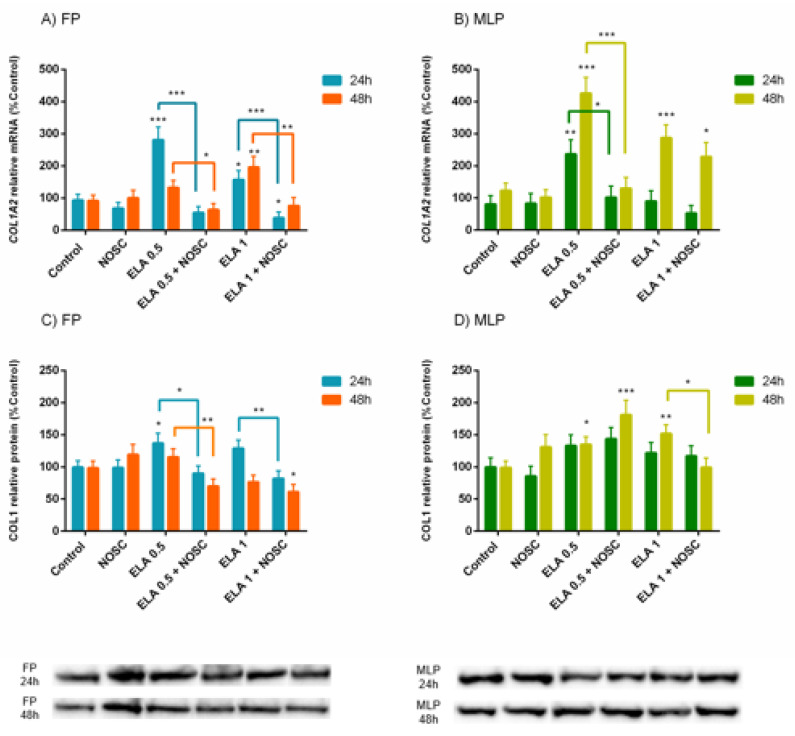
Effect of elastase (ELA; 0.5 or 1 µg/mL), noscapine (NOSC; 45 µg/mL), or ELA (0.5 or 1 µg/mL) + NOSC (45 µg/mL) treatments during 24 or 48 h in explants of mare endometrium from follicular phase (FP) or mid-luteal phase (MLP) on relative collagen type I alpha 2 chain (*COL1A2*) mRNA transcription (**A**,**B**) and collagen type I (COL1) protein relative abundance (**C**,**D**). Results were considered significant at *p* < 0.05 and shown as least square mean ± SEM. Asterisks alone represent significant differences relative to the respective control and asterisks above connecting lines indicate significant differences of ELA + NOSC treatment relative to the respective ELA-treated group (* *p* < 0.05; ** *p* < 0.01; *** *p* < 0.001).

The explants treatment with ELA 0.5 µg/mL increased *COL1A2* transcription in the follicular phase (FP) at 24 h (*p* < 0.001; Figure 2A) or in the mid-luteal phase (MLP) at both treatment times (24 h: *p* < 0.01, 48 h: *p* < 0.001; Figure 2B), comparing to the respective control set. However, the addition of NOSC to ELA 0.5 µg/mL treatment decreased the *COL1A2* mRNA transcription induced by ELA 0.5 µg/mL both in FP (24 h: *p* < 0.001; Figure 2A) and MLP (24 h: *p* < 0.05, 48 h: *p* < 0.001; Figure 2B), in comparison to ELA 0.5 µg/mL respective groups. In FP at 48 h, a downregulation of *COL1A2* mRNA transcription with the combination of ELA 0.5 µg/mL + NOSC treatment was also detected when compared to ELA 0.5 µg/mL-treated group, which did not increase the transcription relative to control group (*p* < 0.05; Figure 2A).

In the Western blot analysis, it was also found that ELA 0.5 µg/mL could raise the relative abundance of COL1 protein in FP at 24 h (*p* < 0.05; Figure 2C), an effect that was reduced by NOSC (*p* < 0.05; Figure 2C). In FP, at 48 h, a decrease in COL1 protein relative abundance with ELA 0.5 µg/mL + NOSC treatment was also found, compared to ELA 0.5 µg/mL group, which was not augmented in relation to its respective control (*p* < 0.01; Figure 2C). In MLP at 48 h, ELA 0.5 µg/mL increased COL1 protein relative abundance (*p* < 0.05; Figure 2D), but no inhibitory effect of NOSC was detected since in the combined treatment of ELA 0.5 µg/mL + NOSC, COL1 relative protein remained increased in relation to the control set (Figure 2D).

The ELA 1 µg/mL treatment augmented *COL1A2* mRNA transcription in the FP at both treatment times (24 h: *p* < 0.05; 48 h: *p* < 0.01; Figure 2A), and NOSC was able to inhibit this effect (24 h: *p* < 0.001; 48 h: *p* < 0.01; Figure 2A). Furthermore, ELA 1 µg/mL + NOSC treatment in FP at 24 h decreased COL1A2 mRNA transcription, with respect to control explants (*p* < 0.05; Figure 2A). In MLP, increased COL1A2 mRNA levels induced by ELA 1 µg/mL were only noted in the longest treatment time (*p* < 0.001; Figure 2B), relative to the respective control groups. However, in MLP, NOSC showed to be ineffective at reducing *COL1A2* transcription induced by ELA 1 µg/mL once it remained increased in comparison to control (*p* < 0.05; Figure 2B).

In FP at 24 h, the combination of ELA 1 µg/mL + NOSC impaired COL1 protein relative abundance in relation to ELA 1 µg/mL-treated group (*p* < 0.01; Figure 2C). Although, in this situation, ELA 1 µg/mL-COL1 protein relative abundance was not augmented with respect to control group (Figure 2C). Besides, the COL1 protein relative abundance was found downregulated with ELA 1 µg/mL + NOSC treatment in FP at 48 h when compared to control group (*p* < 0.05; Figure 2C). The highest concentration of ELA also raised protein relative abundance of COL1 in MLP at 48 h regarding the control group (*p* < 0.01; Figure 2D), but it was inhibited by NOSC treatment (*p* < 0.05; Figure 2D).

Noscapine treatment also differed from other performed treatments. However, to simplify the graph reading, these differences were not signaled in Figure 2. These results are described in Supplementary Table S1. For both *COL1A2* mRNA transcription and COL1 protein relative abundance, no differences were found between NOSC and control groups. Listed in Supplementary Tables S2 and S3 are the differences found in the same treatments between 24 and 48 h of treatment, within each estrous cycle phase, and the differences found in the same treatments between FP and MLP, within each treatment time, respectively.

### 2.3. The Overall Effect of ELA and NOSC Treatments on COL1 in Equine Endometrial Explants

**Figure 3:**
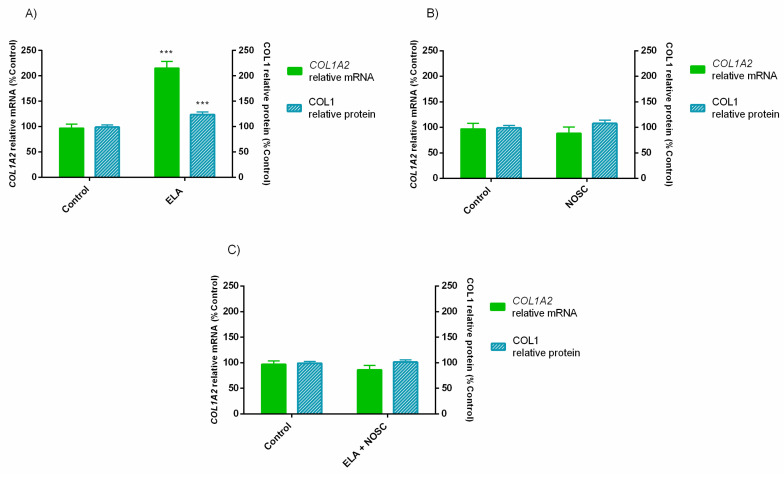
Effect of elastase (ELA; 0.5 and 1 µg/mL) (**A**), noscapine (NOSC; 45 µg/mL) (**B**) and ELA (0.5 and 1 µg/mL) + NOSC (45 µg/mL) (**C**) on relative collagen type I alpha 2 chain (*COL1A2*) mRNA transcription and collagen type I (COL1) protein relative abundance in mare endometrial explants. The results are independent of ELA concentration, estrous cycle phase and treatment time. Results are displayed as least square means ± SEM, and considered significant at *p* < 0.05. Asterisks represent significant differences relative to the respective control (*** *p* < 0.001).

To investigate the overall effects of ELA, NOSC or ELA + NOSC treatments, the ELA concentration, estrous cycle phase and treatment time influences were disregarded. In this analysis it was assumed that ELA concentration, estrous cycle phase and treatment time did not influence *COL1A2* transcription nor COL1 relative protein abundance. The statistical analysis revealed that ELA treatment increased *COL1A2* relative mRNA transcription and COL1 protein relative abundance (*p* < 0.001; Figure 3A). Nevertheless, NOSC treatment did not change *COL1A2* relative mRNA transcription nor COL1 protein relative abundance comparing to the control group (Figure 3B). The combined treatment of ELA + NOSC turn the levels of COL1 expression equal to the control, not differing from it (Figure 3C).

### 2.4. Noscapine Inhibition of the Profibrotic Effects of ELA Regardless of Estrous Cycle Phase and Treatment Time

**Figure 4:**
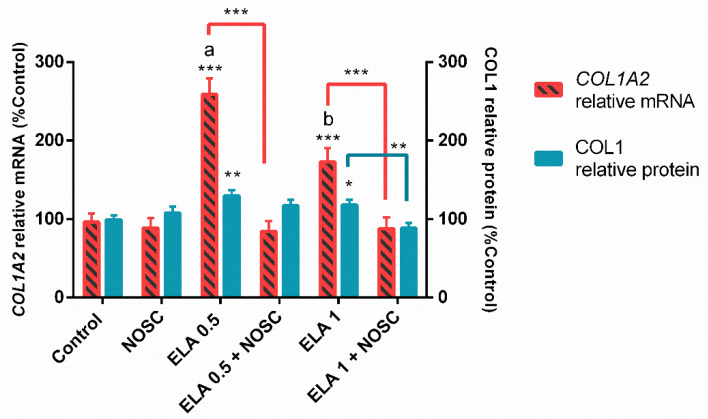
Noscapine (NOSC; 45 µg/mL) inhibition of the effects of elastase (ELA; 0.5 or 1 µg/mL) treatments on relative collagen type I alpha 2 chain (*COL1A2*) mRNA transcription and collagen type I (COL1) protein relative abundance in explants of mare endometrium, irrespective of estrous cycle phase and time of treatment. Results are shown at least square means ± SEM, and considered significant at *p* < 0.05. Different superscript letters indicate significant differences between ELA concentrations (a, b: ELA 0.5 µg/mL ≠ ELA 1 µg/mL; *p* < 0.01). Asterisks alone represent significant differences relative to the respective control and asterisks above the connecting lines indicate significant differences between treatments (* *p* < 0.05; ** *p* < 0.01; *** *p* < 0.001).

Both used concentrations of ELA were capable of increasing *COL1A2* relative mRNA transcription (*p* < 0.001; Figure 4) and COL1 protein relative abundance (ELA 0.5 µg/mL: *p* < 0.01; ELA 1 µg/mL: *p* < 0.05; Figure 4). However, ELA 0.5 µg/mL showed to increase the most *COL1A2* transcription (*p* < 0.01; Figure 4). The inhibitory action of NOSC on ELA 0.5 µg/mL effects in explants of mare endometrium was only detected on *COL1A2* transcripts (*p* < 0.001; Figure 4). Nevertheless, NOSC inhibition of ELA 1 µg/mL effect was effective at both *COL1A2* transcription (*p* < 0.001; Figure 4) and COL1 protein relative abundance (*p* < 0.01; Figure 4).

### 2.5. The Effect of ELA and NOSC Treatments on Mare Endometrial Explants Is Dependent on Estrous Cycle Phase

**Figure 5:**
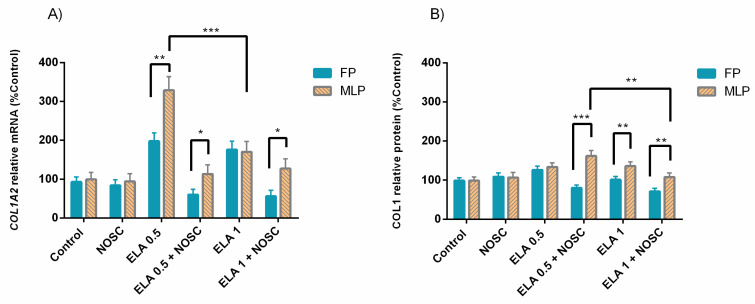
Effect of elastase (ELA; 0.5 or 1 µg/mL), noscapine (NOSC; 45 µg/mL), or ELA (0.5 or 1 µg/mL) + NOSC (45 µg/mL) treatments on relative collagen type I alpha 2 chain (*COL1A2*) mRNA transcription (**A**) and collagen type I (COL1) protein relative abundance (**B**) in explants of mare endometrium from follicular (FP) or mid-luteal (MLP) phases, regardless of treatment time. Results are shown at least square means ± SEM and considered significant at *p* < 0.05. Asterisks above connecting lines indicate significant differences of the same treatment between estrous cycle phase (* *p* < 0.05; ** *p* < 0.01; *** *p* < 0.001).

In MLP, ELA 0.5 µg/mL treatment increased *COL1A2* transcription compared to ELA 1 µg/mL (*p* < 0.001; Figure 5A). Moreover, in MLP, ELA 0.5 µg/mL increased *COL1A2* mRNA transcription more than in FP (*p* < 0.01; Figure 5A). However, the inhibitory effect of NOSC on both ELA concentrations was more effective at reducing *COL1A2* transcription in FP (*p* < 0.05; Figure 5A).

The COL1 protein relative abundance was higher with ELA 1 µg/mL treatment in MLP comparing to FP (*p* < 0.01; Figure 5B). Nevertheless, as for *COL1A2* mRNA transcription, NOSC reduced COL1 protein relative abundance induced by both ELA 0.5 µg/mL and ELA 1 µg/mL more efficiently in FP than MLP (Ela 0.5 µg/mL + NOSC: *p* < 0.001; ELA 1 µg/mL + NOSC: *p* < 0.01; Figure 5B). In MLP, the NOSC-reduction of protein relative abundance of COL1 induced by ELA 1 µg/mL showed to be more effective than reducing COL1 induced by ELA 0.5 µg/mL (*p* < 0.01; Figure 5B).

### 2.6. The Effect of ELA and NOSC Treatments on Mare Endometrial Explants Is Dependent on Treatment Time

**Figure 6:**
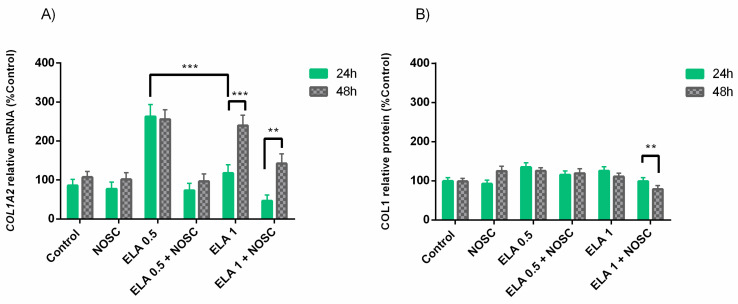
Effect of elastase (ELA; 0.5 or 1 µg/mL), noscapine (NOSC; 45 µg/mL), or ELA (0.5 or 1 µg/mL) + NOSC (45 µg/mL) treatments on relative collagen type I alpha 2 chain (*COL1A2*) mRNA transcription (**A**) and collagen type I (COL1) protein relative abundance (**B**) in equine endometrial explants treated for 24 or 48 h, regardless of estrous cycle phase. Results are shown as least square means ± SEM and considered significant at *p* < 0.05. Asterisks above connecting lines indicate significant differences of the same treatment between time of treatment (** *p* < 0.01; *** *p* < 0.001).

At 24 h of treatment, ELA 0.5 µg/mL induced *COL1A2* transcripts more than ELA 1 µg/mL (*p* < 0.001; Figure 6A). Conversely, at 48 h, ELA 1 µg/mL treatment increased the transcription of *COL1A2* comparing to 24 h (*p* < 0.001; Figure 6A). However, the inhibition of NOSC on ELA 1 µg/mL-induced *COL1A2* was higher at 24 h than at 48 h (*p* < 0.01; Figure 6A).

The analysis of COL1 protein relative abundance revealed that the inhibition of NOSC on COL1 induced by ELA 1 µg/mL was higher at 24 h, when compared to 48 h (*p* < 0.01; Figure 6B).

## 3. Discussion

The proteases (ELA and CAT) and peroxidase (MPO) found in NETs were recently shown to induce COL1 expression in equine endometrium, suggesting NETs enzymes involvement in the pathophysiology of equine endometrial fibrosis [9,11,12,13,14]. However, not only equine endometrosis seems to be affected by profibrotic effects of NETs. Hepatic [47], cardiac [48] and renal [49] fibrosis was also linked to NETs persistence. Recently, NETs were found in the lungs of a cystic fibrosis mouse model in the absence of bacterial infection [50].

In our previous study, equine endometrial explants classified as I/IIA and IIB/III Kenney and Doig [1] categories were treated with ELA 0.5 or 1 µg/mL, and both concentrations induced COL1 expression [9]. Moreover, mare endometrial explants of categories IIA/IIB showed to be more susceptible to profibrotic effect of ELA 0.5 µg/mL at 24 h, while ELA 1 µg/mL only increased *COL1A2* mRNA transcription at 48 h of treatment [11]. In the present study, ELA 0.5 µg/mL induced COL1 expression in endometrial explants, rather than ELA 1 µg/mL (Figure 4). It seems that the response to ELA treatment did not follow a dose–response linear pattern, as its effects did not increase with the highest ELA concentration used. In several biological systems, similar dose–response effects, known as non-monotonic effects, have been reported [51,52]. Low doses of the substance stimulate the expression of more receptors increasing the responses, while high doses inhibit the receptors resulting in decreased responses [53]. This effect is especially noticed in hormone-dependent substances or complex pathways [52]. Endometrosis is a complex disorder and presumed to be controlled by several intricate vias. Therefore, it is likely that the highest dose of ELA tested in the present study was less effective by acting as a non-monotonic substance.

The endometrial explants from both estrous cycle phases reacted to ELA profibrotic action (Figure 2). These results agree with our previous studies, where ELA augmented *COL1A2* transcription [12] and COL1 protein relative abundance [9] in equine endometrial explants, regardless of estrous cycle phase and fibrosis category. However, in the present study the increase of COL1 relative protein abundance was higher in MLP than in FP (Figure 5). In MLP, under the influence of P4, the neutrophil influx to the uterine lumen decreases, impairing uterine defense mechanisms’ efficiency [54]. In addition, the reduced uterine clearance caused by cervix closure predisposes to persistent infectious endometritis [55]. These chronic stimuli may be the cause for establishing a pathological increase in COL1 production in the endometrium. In contrast, in the FP, estrogens increase uterine blood flow, as well as the immune response [54], turning FP more efficient in fighting microorganisms, and therefore decreasing chronicity.

In a shorter treatment time (24h), *COL1A2* mRNA transcription was more influenced by ELA 0.5 µg/mL, whereas at a prolonged treatment period ELA 1 µg/mL dominated the profibrotic response of endometrial explants (Figure 6). Nevertheless, treatment time appeared to have no influence on COL1 protein relative abundance output. Although, the major effect of ELA 1 µg/mL on *COL1A2* mRNA transcription observed at 48 h does not necessarily mean a higher COL1 protein production. The COL1 turnover is dependent on how fast it is produced and degraded [56]. Additionally, collagen protein needs 5000 times more mRNA than a regular protein to result in a considerable amount of deposited collagen [57].

To the best of our knowledge, this is the first investigation that evaluated the in vitro inhibitory effect of NOSC on ELA induced COL1 in equine endometrium. Our results showed that NOSC could reduce COL1 expression (induced by ELA; Figure 3) in equine endometrial explants at both estrous cycle phases and times of treatment (Figure 2). Endometrosis treatment has been challenging, with no effective treatment found so far. To make progress on endometrosis treatment, we have also shown that ELA, CAT or MPO fibrotic effect on the mare endometrium can be inhibited in vitro by sivelestat sodium salt, β-keto-phosphonic acid, or 4-aminobenzoic acid hydrazide, respectively [12,13,14]. These promising results are the latest advances in equine endometrosis putative treatment. However, endometrosis is characterized by many triggering factors. This way, a single drug capable of inhibiting more than one etiological factor will facilitate the therapeutic approach.

Noscapine is a safe drug with little to no toxicity, even if it is administered at supra-pharmacological doses [41]. It also does not affect the natural immune response [46]. The mechanism by which NOSC acts as an anticancer molecule is still currently under study. Noscapine causes cancer cell apoptosis and binds to tubulin [58]. The binding to tubulin alters its conformation and attenuates microtubules, without affecting the total tubulin polymer mass in cells. The microtubules stay longer in a paused mode leading to a block in mitosis at prometaphase, thus inducing apoptosis of neoplasic cells [58,59,60]. Interestingly, benign cells are not affected by NOSC apoptotic effect. The selective anti-neoplasic action of NOSC may be caused by the lack of normal mitotic spindle assembly checkpoint of neoplasic cells that induces mitotic death called “mitotic catastrophe” [60,61]. A recent review of the use of NOSC to treat glioblastoma shows the putative pathways involved in the mechanism of action of noscapine to treat glioblastoma [58]. In glioma cell lines, NOSC inhibited the hypoxia-inducible factor-1 (HIF-1) pathway that is related to angiogenesis and cancer severity by reducing HIF-1 gene and vascular endothelial growth factor (VEGF). Thus, NOSC could be used as an antiangiogenic chemotherapy for glioma [62]. The same group also associated the NOSC induction of apoptosis with activation of the c-jun N-terminal kinase signaling (JNK) pathway, simultaneously with inactivation of the extracellular signal regulated kinase (ERK) signaling pathway and phosphorylation of the antiapoptotic protein Bcl-2 [60]. The NOSC anticancer activity seems to be not only related to angiogenesis, and cell proliferation, but also to the inhibition of nuclear factor kappa-light-chain-enhancer of activated B cells (NF-kB) pathway [63,64]. The NF-kB pathway is involved in immune response, but its dysregulation is also linked to inflammatory and cancer disorders. In human leukemia and myeloma cells, NOSC inhibited NF-kB through NF-kB kinase turning the neoplasic cells sensitive to tumor necrosis factor and chemotherapy [63].

Noscapine was also described for the treatment and prevention of infectious diseases [65,66]. This alkaloid blocks the movement of microorganisms within the host cells by inhibiting the cytoplasmic transport. In addition, NOSC binds to tubulin receptor preventing the viruses’ access to the cytoskeleton, and thus avoiding viral amplification [65]. Recently, computational studies showed that NOSC could be used for COVID-19 treatment, because the molecule binds to the main protease of the virus responsible for virus replication [46].

To the best of our knowledge, only a study has reported the action of NOSC as an antifibrotic drug. In cultured human lung fibroblasts, NOSC reduced transforming growth factor β-induced stress fiber, without affecting the microtubule content [45]. Additionally, it was also demonstrated that prostaglandin E2 receptor (EP2) mediated the activation of protein kinase A (PKA) that is the putative mechanism responsible for noscapine’s antifibrotic activity in pulmonary fibroblasts [45].

The present study did not investigate the NOSC mechanism of action in equine endometrial explants. Nevertheless, it has been suggested that NOSC, by binding to microtubules, also inhibits myofibroblast differentiation [67]. During endometrosis, myobfibroblasts (differentiated from fibroblasts) are the main cells that synthesize fibers of COL and other components of the extracellular matrix [2,8]. Interestingly, since the neoplasic and fibrotic processes may share similar signaling pathways, NOSC mechanisms of action on fibrosis therapy, might be the same present in cancer cells. For example, HIF-1α stimulates excessive extracellular matrix deposition in fibrosis [68], and since NOSC is capable of inhibiting HIF-1, one might suggest this pathway could be a new therapeutic target.

NF-kB is a pathway involved in the immune response [69] to microorganisms that reach the uterus, by activating proinflammatory cytokines [70]. In mares susceptible to post-breeding endometritis, the proinflammatory cytokines remain upregulated after 24 h post-breeding, contrary to resistant mares [71]. The long-standing endometritis contributes to the severity, progression, and irreversibility of endometrosis alterations [2]. Since NOSC inhibits the NF-kB pathway [63], its use in equine endometrosis might be a hopeful treatment especially in mares susceptible to post-breeding endometritis to prevent proinflammatory cytokine prolonged increase.

We demonstrated the protective effect of prostaglandin (PG)E_2_, through EP2 receptor, in equine endometrial explants treated with NETs enzymes [10]. In equine endometrial tissues stimulated with ELA, high COL1 production was associated with impairment of PGE_2_ or EP2 transcripts [10]. The inhibition of ELA with sivelestat induced PGE_2_ production by equine endometrial explants, suggesting the antifibrotic action of PGE_2_ in equine endometrosis [11]. Although this study used a different molecule to inhibit ELA, the inhibition of ELA might activate the protective effect of PGE_2_. Furthermore, in vitro preconditioning of equine adipose mesenchymal stem cells with PGE_2_, acting through its receptor EP2, enhanced their immunomodulatory competence and has been suggested for use in the treatment of fibrotic diseases [35]. According to Kach et al. [45], NOSC acted through EP2 receptor in human lung fibroblasts. Thus, we speculate that a similar noscapine’s antifibrotic activity may be involved in mare endometrial tissues. However, further studies on the expression of this receptor must be carried out to evaluate its involvement in equine endometrial explants response to NOSC treatment.

## 4. Materials and Methods

### 4.1. Mare Sample Collection

According to the legislation of Europe (EFSA, AHAW/04–027) and inspected by the official veterinary, healthy cyclic mares were euthanized at a slaughterhouse in Rawicz, Poland, and their uteri were collected within 15 min, and transported to the laboratory on ice. The average age of mares used in the present study was 12 ± 3 years old (mean ± SD). Jugular blood samples were also collected into ethylenediaminetetraacetic acid (EDTA) tubes for progesterone (P4) assays. In the laboratory, determination of mare estrous cycle phase was performed by ovarian and uterine assessment, and further confirmed by plasma P4 concentration. In the follicular phase (FP), mares presented plasma P4 concentration < 1 ng/mL, and a follicle > 35 mm diameter. In the mid-luteal phase (MLP), mares presented P4 plasma concentration > 6 ng/mL, a well-developed corpus luteum and follicles between 15 and 20 mm in diameter [72]. The samples were also evaluated for the presence of endometritis, as previously described [9,73]. Briefly, the inspection of increased mucus production or altered coloration of endometrium surface, as signs of endometritis was done macroscopically. Endometrial cells obtained with a sterile cotton swab, which was rolled on a glass slide, were stained with Diff-Quick and examined under a light microscope for the presence of bacteria and/or neutrophils [9,73]. Only the endometria that showed no signs of endometritis were considered for this study. The uteri from FP (*n* = 8) and MLP (*n* = 7) were transported on ice to the laboratory and placed in ice-cold Dulbecco’s modified Eagle’s medium (DMEM) F-12 Ham medium (D/F medium; 1:1 (*v/v*); D-2960; Sigma-Aldrich, St Louis, MO, USA), supplemented with 2 µg/mL amphotericin (A2942; Sigma-Aldrich, St Louis, MO, USA), 100 µg/mL streptomycin (S9137; Sigma-Aldrich, St Louis, MO, USA) and 100 IU/mL penicillin (P3032; Sigma-Aldrich, St Louis, MO, USA).

### 4.2. Equine Endometrial Explants In Vitro Culture

The endometrial explants were collected and prepared, as previously described [12]. Briefly, the collected uteri were washed in phosphate-buffered saline (PBS) with 100 μg/mL streptomycin (S9137; Sigma, St Louis, MO, USA) and 100 IU/mL penicillin (P3032; Sigma, St Louis, MO, USA). After, strips of endometria were detached with scissors from the underlying myometrium, from the uterine horn ipsilateral to the active ovary, and placed in PBS supplemented with 2 µg/mL amphotericin (A2942; Sigma-Aldrich, St Louis, MO, USA), 100 µg/mL streptomycin (S9137; Sigma-Aldrich, St Louis, MO, USA) and 100 IU/mL penicillin (P3032; Sigma-Aldrich), in a petri dish on ice. The explants weighting 20–30 mg were then placed in 24-well cell culture sterile plates (Eppendorf, #0030 722.116) with culture medium and gentle shaking (150 rpm) during 1 h at 38 °C and 5% CO_2_ in a humidified atmosphere chamber (Biosafe Eco-Integra Biosciences, Chur, Switzerland), as preincubation. The DMEM culture medium was supplemented, as follows: (i) 2 µg/mL amphotericin (A2942; Sigma-Aldrich, St Louis, MO, USA), (ii) 100 IU/mL penicillin (P3032; Sigma-Aldrich, St Louis, MO, USA), (iii) 100 µg/mL streptomycin (S9137; Sigma-Aldrich, St Louis, MO, USA) and (iv) 0.1% (*w*/*v*) bovine serum albumin (BSA; 735078; Roche Diagnostics, Mannheim, Germany). After the pre-incubation, culture media were replaced and the explants treated in quadruplicate for 24 or 48 h, as follows: (i) vehicle (negative control)—culture medium; (ii) ELA (0.5 µg/mL or 1 µg/mL; A6959, Applichem GmbH, Darmstadt, Germany); (iii) noscapine hydrochloride hydrate (NOSC; 45 µg/mL; N9007; Merck, Darmstadt, Germany); (iv) ELA (0.5 µg/mL or 1 µg/mL) + NOSC (45 µg/mL); or (v) oxytocin (OXT; 10-7 M; O3251; Sigma-Aldrich, St Louis, MO, USA), used as a positive control for PGF2α secretion [74,75]. Noscapine treatment was performed at the stage of culture media replacement, to give time for NOSC to bind, whereas the ELA treatment was achieved 1 h later. The concentrations of the treatments used were based on other studies. Elastase 0.5 and 1 µg/mL were already proved to induce COL1 expression in equine endometrial explants [9,11]. Noscapine was determined by the optimum concentration, which was able to inhibit *COL1A2* transcription, in a dose–response trial (0.45, 4.5, 45, 450 and 4500 µg/mL) supported by the in vitro studies of Yang et al. [76] and Kach et al. [45]. The explants were collected in RNAlater^®^ (R901, Sigma-Aldrich, St Louis, MO, USA), and culture media in 1% PG stabilizer solution (0.3 M EDTA, E5134, Sigma-Aldrich + 1% aspirin, A2093, Sigma-Aldrich, St Louis, MO, USA), and stored at −80 °C.

**Figure 7:**
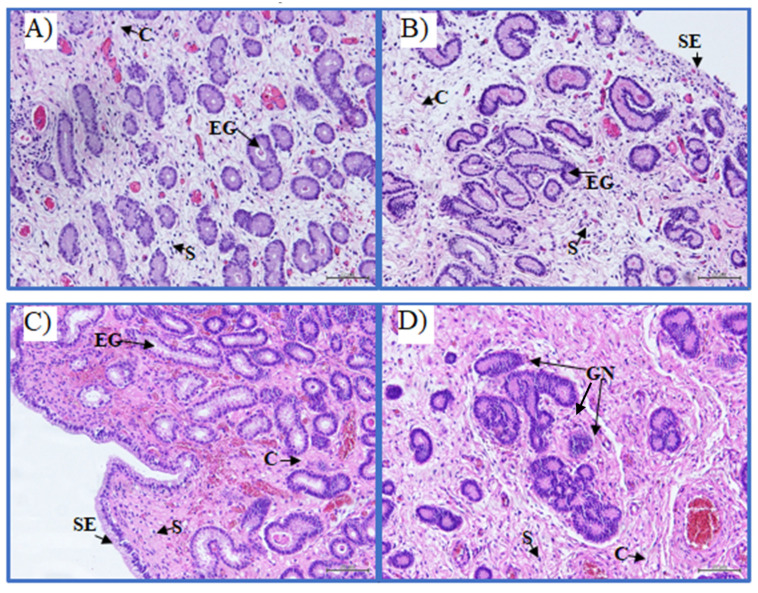
Representative equine endometrial biopsies classified as category IIA (**A**,**B**) and IIB (**C**,**D**) according to Kenney and Doig (1986) classification. Endometrial glands (EG), glandular nests (GN), collagen fibers (C), stromal cells (S), surface epithelium (SE). Staining with hematoxylin and eosin. Scale bar = 20 μm.

To perform the endometrial histopathological classification [1], two pieces of endometrial tissue from the ipsilateral horn of the ovary that showed the dominant structure were collected in 4% buffered paraformaldehyde. In this study, only the samples of categories IIA and IIB, corresponding to mild (IIA) and to moderate (IIB) histopathological lesions were used (Figure 7) [1].

### 4.3. Determination of Mare Endometrial Explants Viability

To evaluate explant viability, PGF_2α_ secretion in conditioned culture media and LDH activity were determined, as previously described [12].

The explant secretory capacity was evaluated by measuring the amount of PGF_2α_ secreted to the culture media using an enzyme immunoassay kit, according to the instructions provided by the manufacturer (PGF2α ELISA kit—ADI-901-069, Enzo, New York, NY, USA). The standard curve ranged from 3 to 50,000 pg/mL and intra- and interassay coefficients of variation were 5.9% and 4.3%, respectively. Since live cells secrete PGF_2α_ in response to OXT, an increase in the concentration of PGF_2α_ in culture media, is a good estimate of cell secretory capacity/viability [74,75].

Lactate dehydrogenase activity allows the evaluation of membrane integrity of viable cells. If the cell membrane is injured, LDH is liberated to the extracellular space. The LDH activity was measured using a colorimetric assay kit, as indicated by the producer’s protocol (ab102526, Abcam, UK), extracellularly in conditioned culture media (1:100 diluted in kit assay buffer) and intracellularly in 10 mg explants incubated for 1, 24 and 48 h. The incubated explants were macerated (TissueLyser II; Qiagen, Madrid, Spain) in 250 μL of kit assay buffer and then diluted 1:200 times in kit assay buffer. The activity of LDH was read in a kinetic mode at 450 nm wavelength, at 37 °C, for 1 h using a spectrophotometer (FLUOstar OPTIMA Microplate Reader; BMG Labtech; Ortenberg, Germany). Thus, the viability of the explants was calculated from the quotient of the intracellular LDH activity and the total activity (extracellular plus intracellular LDH) [77].

### 4.4. Molecular Biology: Extraction of Total RNA, cDNA Synthesis and Quantitative Real-Time Polymerase Chain Reaction (qPCR) for COL1A2 mRNA Determination

The total RNA was extracted from treated explants using TRI Reagent^®^ (T9424; Sigma-Aldrich, St Louis, MO, USA) according to the producer´s instructions. The Nanodrop system (ND 200C; Fisher Scientific, Hamton, PA, USA) was used for RNA quantification. The RNA quality was evaluated by electrophoresis of the RNA in a 1.5% agarose gel, and red staining (41,003; Biotium, Hayward, CA, USA) enabling the visualization of 28S and 18S rRNA bands. The synthesis of cDNA was undertaken using reverse transcriptase enzyme (M5313; Promega; Madison, WI, USA) and oligo (dT) primer (C1101; Promega; Madison, WI, USA) from 1000 ng total RNA in a 20 μL reaction volume.

The validation of the reference gene ribosomal protein L32 (*RPL32*), as well as the sequences for the target gene *COL1A2* and reference gene (Table 2), were previously determined [12,78]. Both genes were run in duplicate at the same plate (96-well plate; 4306737; Applied Biosystems) in a StepOnePlus™ Real-Time PCR System (Applied Biosystems, Warrington, UK). The qPCR products specificity was achieved, as referred [12,79]. A non-template control was run in all plates for all genes, omitting cDNA sample, as a control of extraneous nucleic acid contamination and primer dimer formation.

### 4.5. Western Blot: COL1 Protein Relative Abundance Determination

The explants were prepared to perform the Western blot analysis to evaluate the relative protein abundance of COL1 by the nonstaining total protein loading control method, as reported [12]. Briefly, explants were disrupted using TissueLyser II (Qiagen, Madrid, Spain) in ice-cold RIPA buffer (50 mM Tris-HCl, pH 7.4, 50 mM EDTA, 150 mM NaCl, and 1% Triton X-100) supplemented with a protease inhibitor (cOmplete Mini Protease Inhibitor Cocktail Tablets, 1 tablet per 10 mL of buffer; Roche, Basel, Switzerland). The protein concentration was quantified using Bradford reagent (500-0006; Bio-Rad, Hercules, CA, USA), 30 μg of protein in 2× Laemmli Loading Buffer (62.5 mM Tris-HCl, pH 6.8 containing 2% SDS, 25% glycerol, 0.01% bromophenol blue) and DTT 50 mM were denatured at 95 °C for 5 min, and then cooled on ice for 10 min. Then, the samples were loaded on an 8% acrylamide gel (MB04501; Nzytech, Lisbon, Portugal) with 0.5% (*v*/*v*) 2,2,2-trichloroethanol (808610; Merck, Darmstadt, Germany) incorporated in gel using a Mini-PROTEAN^®^ Vertical Tetra Cell system (Bio-Rad, Hercules, CA, USA). In a single lane of all gels, a standard endometrial sample (30 μg) was also loaded to perform the band normalization and to compare gels. After running, the gels were transferred to a nitrocellulose membrane (GE10600001; Amersham™ Protran^®^ Western blotting membranes, nitrocellulose pore size 0.2 μm, roll W × L 300 mm × 4 m; GE Healthcare; Chicago, IL, USA). The membranes were exposed during 1 min to UV light at ChemiDoc XRS + System (Bio-Rad, Hercules, CA, USA) to obtain normalization image. The COL1 primary antibody was purchased from Novotec (20121; RRID: AB_2891017; Novotec, Lyon, France), diluted 1:1000 and incubated at 4 °C overnight. Since it is an antibody from bovine origin, it was previously validated to the horse tissues by Rebordão et al. [9] using bovine skin and equine endometrium and skin in the same membrane. This previous validation [9] revealed that all the bovine and equine samples showed a band near 126 kDa, which is the predicted size for collagen type I alpha 2 chain [80]. In the present study, no alteration on the molecular weight band was detected, so it is assumed that the band around 126 kDa corresponds to collagen type I alpha 2 chain in equine endometrial explants, as previously demonstrated [9]. After, the secondary antibody Horseradish peroxidase (HRP)-conjugated anti-rabbit (P0448; RRID: AB_2617138; DakoCytomation, Carpinteria, CA, USA) was diluted 1:20,000 and incubated at room temperature during 1.5 h. Luminol enhanced chemiluminescence (Super Signal West Pico, 34077; Thermo Scientific, Waltham, MA, USA) was used for COL1 protein relative abundance bands detection, which were analyzed in Image Lab 6.0 (Bio-Rad, Hercules, CA, USA) software by a multichannel protocol detecting the total protein lanes in stain-free total protein membrane image and COL1 bands on chemiluminescence image [81]. The amount of the COL1 protein was calculated by a factor of normalization to adjust the variability of the loaded protein [12,81].

### 4.6. Statistical Data Analysis

Initially, the data normality was assessed by visualization and by the test of Kolmogorov–Smirnov using Proc Univariate of SAS v. 9.4 (SAS Institute Inc., Cary, NC, USA).

The one-way analysis of variance (ANOVA) followed by Tukey’s multiple comparisons test (GraphPAD PRISM, Version 6.00, 253 GraphPad Software, San Diego, CA, USA) was the method used to analyze viability data. These results are displayed as mean ± SEM and significant set at *p* < 0.05.

Once *COL1A2* transcription and COL1 protein relative abundance variables did not show a normal distribution, the square root was used to convert the data. These data were further analyzed, firstly by the PROC GLM (SAS v. 9.4; SAS Institute Inc., Cary, NC, USA) to obtain the response of *COL1A2* mRNA and COL1 protein relative abundance to the different treatments (combination of the concentration of ELA, the effect of NOSC, estrous cycle phase, and time of treatment) causing 24 treatment combinations. Afterwards, the PDIFF of PROC GLM (SAS v. 9.4; SAS Institute Inc., Cary, NC, USA) evaluated the comparison of the least square means obtained from the combined treatments. The two-, three- and four-way interactions were examined, as well. The results were considered significant at *p* < 0.05. The obtained least square means were back transformed. The graphical presentation was made in GraphPAD PRISM (Version 6.00, 253 GraphPad Software, San Diego, CA, USA), and shown as least square means ± SEM.

## 5. Conclusions

The profibrotic effect of ELA in IIA/IIB [1] equine endometrial explants was inhibited in vitro by NOSC treatment. These findings might contribute for the future development of novel endometrosis therapy. In vitro studies are limited, and future in vivo studies should be carried out to confirm our preliminary in vitro results. Other NETs components, such as CAT and MPO, already demonstrated to act as profibrotic agents in equine endometrial explants in an in vitro model. Further studies, investigating the effect of NOSC on both CAT and MPO, will determine if NOSC could be used as one single inhibitor capable of inhibiting all the profibrotic enzymes present in NETs.

## Figures and Tables

**Table 1:** Activity of lactate dehydrogenase (LDH) at 1, 24 and 48 h of explant incubation, as an indicator of explant viability. Since damaged cells release LDH extracellularly, the viability was measured by the calculation of quotient of the intracellular LDH activity and the total activity (extracellular plus intracellular LDH). Data are shown as means ± SEM. Statistical differences between times of incubation are signaled by different superscript letters (a,b and b,c: *p* < 0.05; a,c: *p* < 0.01).

Incubation Period	Activity of LDH (%)
1 h	95.1 ± 0.7 ^a^
24 h	90.7 ± 0.7 ^b^
48 h	87.6 ± 1.0 ^c^

**Table 2:** Sequences of primers utilized in quantitative real-time polymerase chain reaction (qPCR).

Gene (Accession Number)	Sequence 5′-3′	Amplicon
*RPL32* (XM_001492042.6)	Forward: AGCCATCTACTCGGCGTCA	144
	Reverse: GTCAATGCCTCTGGGTTTCC	
*COL1A2* (XM_001492939.3)	Forward: CAAGGGCATTAGGGGACACA	196
	Reverse: ACCCACACTTCCATCGCTTC	

*RPL32*—ribosomal protein L32, *COL1A2*—collagen type I alpha 2 chain.

## Data Availability

Data will be available upon request to the corresponding author.

## Supplementary Materials

The following are available online at https://www.mdpi.com/article/10.3390/ijms22105333/s1.
