# Supplementary material for: Noscapine Acts as a Protease Inhibitor of In Vitro Elastase-Induced Collagen Deposition in Equine Endometrium
Source: Int J Mol Sci. 2021 May 19;22(10):5333. doi: 10.3390/ijms22105333 (PMC8159119; doi:10.3390/ijms22105333)
Supplement: Supplementary file 1 [file ijms-22-05333-s001.zip › ijms-1205147-supplementary.pdf]

Supplementary Table S1: List of differences found between noscapine (NOSC; 45 µg/mL) treatment, and the other performed treatments: (i) elastase (ELA; 0.5 and 1 µg/mL) or (ii) ELA (0.5 and 1 µg/mL) + NOSC (45 µg/mL) for *COL1A2* transcription and COL1 protein relative abundance in equine endometrial explants from follicular (FP) or mid-luteal (MLP) phases treated for 24h or 48h.

**Evaluated variable**	**Treatment comparison**	**P value**	**Figure**
COL1A2 transcription	NOSC vs ELA 0.5 FP 24h	P < 0.001	2A
	NOSC vs ELA 1 FP 24h	P < 0.01	
	NOSC vs ELA 1 FP 48h	P < 0.05	
	NOSC vs ELA 0.5 MLP 24h	P < 0.01	2B
	NOSC vs ELA 0.5 MLP 48h	P < 0.001	
	NOSC vs ELA 1 MLP 48h	P < 0.001	
	NOSC vs ELA 1 + NOSC MLP 48h	P < 0.01	
COL1 protein relative abundance	NOSC vs ELA 0.5 FP 24h	P < 0.05	2C
	NOSC vs ELA 0.5 + NOSC FP 48h	P < 0.05	
	NOSC vs ELA 1 FP 48h	P < 0.05	
	NOSC vs ELA 1 + NOSC FP 48h	P < 0.01	
	NOSC vs ELA 0.5 MLP 24h	P < 0.05	2D
	NOSC vs ELA 0.5 MLP 24h	P < 0.05	

COL1A2 - collagen type 1 α2; COL1 – collagen type 1; NOSC – noscapine; ELA 0.5 – elastase 0.5 µg/mL; ELA 1 – elastase 1 µg/mL; FP – follicular phase; MLP – mid-luteal phase.

Supplementary Table S2: List of differences found in the same treatments between 24h and 48h of treatment, within each estrous cycle phase.

**Evaluated variables**	**Treatment comparison**	**P value**	**Figures**
COL1A2 transcription	ELA 0.5 24h FP *vs* ELA 0.5 48h FP	P < 0.001	2A
	ELA 0.5 24h MLP *vs* ELA 0.5 48h MLP	P < 0.01	2B
	ELA 1 24h MLP *vs* ELA 1 48h MLP	P < 0.001	
	ELA 1 + NOSC 24h MLP *vs* ELA 1 + NOSC 48h MLP	P < 0.001	
COL1 protein relative abundance	ELA 0.5 + NOSC 24h FP *vs* ELA 0.5 + NOSC 48h FP	P < 0.01	2C
	ELA 1 24h FP *vs* ELA 1 48h FP	P < 0.01	

COL1A2 - collagen type 1 α2; COL 1 – collagen type I; ELA 0.5 – elastase 0.5 µg/mL; ELA 1 – elastase 1 µg/mL; NOSC – noscapine; FP – follicular phase; MLP – mid-luteal phase.

Supplementary Table S3: List of differences found in the same treatments between the follicular phase (FP) and mid-luteal phase (MLP) of the estrous cycle, within each treatment time.

**Evaluated variables**	**Treatment comparison**	**P value**	**Figures**
COL1A2 transcription	ELA 0.5 48h FP *vs* ELA 0.5 48h MLP	P < 0.001	2A, 2B
	ELA 1 + NOSC 48h FP *vs* ELA 1 + NOSC 48h MLP	P < 0.01	
COL1 protein relative abundance	ELA 0.5 + NOSC 48h FP *vs* ELA 0.5 + NOSC 48h MLP	P < 0.001	2C, 2D
	ELA 1 48h FP *vs* ELA 1 48h MLP	P < 0.001	
	ELA 1 + NOSC 48h FP *vs* ELA 1 + NOSC 48h MLP	P < 0.05	

COL1A2 - collagen type 1 α2; COL 1 – collagen type I; ELA 0.5 – elastase 0.5 µg/mL; ELA 1 – elastase 1 µg/mL; NOSC – noscapine; FP – follicular phase; MLP – mid-luteal phase.
